# Climate Services to Improve Public Health

**DOI:** 10.3390/ijerph110504555

**Published:** 2014-04-25

**Authors:** Michel Jancloes, Madeleine Thomson, María Máñez Costa, Chris Hewitt, Carlos Corvalan, Tufa Dinku, Rachel Lowe, Mary Hayden

**Affiliations:** 1 Health and Climate Foundation, Washington, DC 20005, USA; 2 International Research Institute for Climate and Society, Columbia University, Palisades, New York, NY 10964, USA; E-Mails: mthomson@iri.columbia.edu (M.T.); tufa@iri.columbia.edu (T.D.); 3 Mailman School of Public Health, Columbia University, New York, NY 10032, USA; 4 Climate Service Center, Fischerstwiete 1, Hamburg 20095, Germany; E-Mail: maria.manez@hzg.de; 5 Met Office UK, Exeter, Devon EX1 3PB, UK; E-Mail: chris.hewitt@metoffice.gov.uk; 6 Pan American Health Organization, Brasilia CEP 70800-400, Brazil; E-Mail: corvalanc@paho.org; 7 Institut Català de Ciènces Del Clima, Barcelona 08005, Spain; E-Mail: rachel.lowe@ic3.cat; 8 National Center for Atmospheric Research, Boulder, CO 80301, USA; E-Mail: mhayden@ucar.edu

**Keywords:** public health, climate services, weather alert, health policies

## Abstract

A high level expert panel discussed how climate and health services could best collaborate to improve public health. This was on the agenda of the recent Third International Climate Services Conference, held in Montego Bay, Jamaica, 4–6 December 2013. Issues and challenges concerning a demand led approach to serve the health sector needs, were identified and analysed. Important recommendations emerged to ensure that innovative collaboration between climate and health services assist decision-making processes and the management of climate-sensitive health risk. Key recommendations included: a move from risk assessment towards risk management; the engagement of the public health community with both the climate sector and development sectors, whose decisions impact on health, particularly the most vulnerable; to increase operational research on the use of policy-relevant climate information to manage climate- sensitive health risks; and to develop in-country capacities to improve local knowledge (including collection of epidemiological, climate and socio-economic data), along with institutional interaction with policy makers.

## 1. Background

Health providers have long understood that knowledge about the climate is relevant to clinical practice and public health programming. Building on recent advances in our understanding of climate science and the risks of climate change, national climate services and related institutions have begun to prioritize the development and provision of global services, to better meet the needs of users and decision makers in the management of climate-related risks.

However, while climate services are being developed for different sectors, such as agriculture and water resource planning, it is clear that effective demand from the health community in many regions is very low or non-existent. Health policy makers and practitioners are well aware of the impact of climate on the dynamics of many diseases and health conditions, such as malaria, emerging infectious diseases, cardiovascular disease, nutrition deficiencies and food security. Despite this understanding, climate information is rarely exploited as a means to help prevent and control such health risks. Further, climate service providers are generally absent from the broad public health community of practice.

## 2. Discussions

Recently, international fora have called for a paradigm shift in the development and delivery of weather and climate services for the health community [1,2]. This builds on innovative “demand led” approaches, which can be integrated into current decision-making processes for both the formal health sector and other sectors that impact human health [3]. 

This solution-driven approach was the subject of a high-level expert panel at the recent Third International Conference of Climate Services (ICCS3), held in in Montego Bay, Jamaica, 4–6 December 2013. The meeting was developed in response to the Global Framework for Climate Services (GFCS), launched in Geneva in 2012 by the World Meteorological Congress. According to WMO [4], “The GFCS is a global partnership of governments and organizations that produce and use climate information and services. It seeks to enable researchers and the producers and users of information to join forces to improve the quality and quantity of climate services worldwide, particularly in developing countries” [5]. Health is one of the four priority areas of focus for the GFCS, the other three being agriculture, water and disaster risk reduction [2].

This panel was organized by the Health and Climate Foundation in collaboration with the German Climate Service Center. After opening remarks from the organizers, and presentations from the UK Met Office, the Pan American Health Organization and the International Research Institute for Climate and Society, an open discussion with workshop participants was held, which addressed the following questions:

- Why is there such a gap between climate and health communities?
- How can we best move towards sustained collaboration?

This Communication summarizes the results of the discussion. Significant challenges to the use of climate information in operational decision-making were observed, including the fact that the health sector makes up only a fraction of decisions which impact human health. Other significant sectors that have a big impact on public health include agriculture, water resource management and disaster risk reduction (which have their own distinct information and decision-making cultures and mechanisms), as well as education and urban planning. Thus, health may be considered a down-stream outcome of many sectors—but a priority of only one. An example of this type of disconnect is the historic focus of agricultural production on yield—with little or no attention to nutrition. It is essential to explicitly highlight the importance of health deliverables for other sectors. At the same time, the contribution of health to the performance of critical national development sectors should be emphasized. A Health exemplar, prepared for the GFCS in collaboration with WHO [2], sets the stage for the engagement of the health sector with GFCS through a “User Interface Platform” but does little to connect a broader public health agenda that builds on the responses of representatives from other key sectors. This is a concern that must be addressed at the global and local level. 

The climate community expressed a clear readiness to provide services for public health authorities and health related issues, through demand-oriented institutional relationships. However, identifying and sustaining long-term partnerships with health policy and practice communities can be challenging. As a prerequisite for sustained collaboration, it was recognized that health officials have to be familiar with the potential use of climate services and information. At the same time, climate service providers have to understand the epidemiological and operational context of problems for health officials and solutions for which climate information can influence their decisions. 

Future requirements include the need for appropriate evidence of the cost/benefit value of climate services to the health community and the inclusion of climate information in health planning and programming. While progress has been made through time-limited projects and training events, the lack of an institutionalized approach to the development of climate services for the health sector was considered a major barrier to a wider uptake. The technologies capable of providing “early warning” are rapidly improving. However, ensuring effective response to such warnings is a challenge and requires political will. 

Despite the limitations noted, good examples of the development and operationalization of weather and climate services for health were presented by the panelists with examples from Brazil [6], Ethiopia [7], Nepal, Niger, United Kingdom and Yemen, among others. References were also made to existing multidisciplinary networks dealing with meningitis epidemics [8] and human/animal leptospirosis (the GLEAN Project).

A particular challenge observed across initiatives was the gap between the time and space scales needed by users *vs.* what can be credibly delivered based on our scientific knowledge and capabilities. There is a rapidly developing capacity (through better modelling and more powerful computers) to accurately forecast short-term weather events and to provide probabilistic seasonal climate forecasts and longer-term climate scenarios. Also, participants pointed out the need for reliable epidemiological data, which are still scarce in many countries, to enable relevant analyses of climate information that will lead to meaningful findings for decision-making by the health community.

Moving forward, priority is being given to opportunities with a high level of pay-off in both a practical and political perspective. Examples proposed included emergency preparedness and prevention and response to acute events such as epidemics or heat waves [9]. Alternatively, incorporating historical and “near real time” monitored climate information into routine planning and assessment processes, to better allocate resources (both in geographic space and for different seasons throughout the year) was considered a significant opportunity. Experience indicates that while many ministries of health are motivated to discuss “climate” in the context of “climate change”, the reality on the ground is that they should prioritize better management of current climate variability, to meet pressing health priorities, above information on longer time horizons. 

An applied systems approach, which seeks to address problems of public health significance is required. This can be achieved through the use of new knowledge and tools that bridge research and decision-maker needs. This applied approach combines hard and soft systems methodology; seeking to optimize the outcomes of the research at the interface of these two systems (climate services and public health).

## 3. Conclusions

Following the panel presentations and discussion, a set of key recommendations for better climate service provision, to ultimately improve public health, emerged:

(1) To move from risk assessment towards risk management by clearly identifying climate information needs for influencing decisions related to climate-sensitive health risk. For example, developing risk maps, warning and alert thresholds and intervention impact analyses.
(2) To ensure not only better engagement of the public health community with the climate sector but also all development sectors whose decisions can have an impact on health, in particular, the health of the most vulnerable populations.
(3) To intensify operational research on the use of policy-relevant climate information—past, present and future—in the better management of climate-sensitive health risks.
(4) To develop in-country capacities for better local knowledge (including collection of epidemiological, climate and socio-economic data), along with institutional interaction with policy makers.
